# Natural History of Malignant Bone Disease in Renal Cancer: Final Results of an Italian Bone Metastasis Survey

**DOI:** 10.1371/journal.pone.0083026

**Published:** 2013-12-30

**Authors:** Daniele Santini, Giuseppe Procopio, Camillo Porta, Toni Ibrahim, Sandro Barni, Calogero Mazzara, Andrea Fontana, Alfredo Berruti, Rossana Berardi, Bruno Vincenzi, Cinzia Ortega, Davide Ottaviani, Giacomo Carteni, Gaetano Lanzetta, Vladimir Virzì, Matteo Santoni, Nicola Silvestris, Maria Antonietta Satolli, Elena Collovà, Antonio Russo, Giuseppe Badalamenti, Stefano Luzi Fedeli, Francesca Maria Tanca, Vincenzo Adamo, Evaristo Maiello, Roberto Sabbatini, Alessandra Felici, Saverio Cinieri, Giuseppe Tonini, Sergio Bracarda

**Affiliations:** 1 Department of Medical Oncology, University Campus Bio-Medico, Rome, Italy; 2 Fondazione IRCCS Istituto Nazionale dei Tumori, Milan, Italy; 3 Fondazione IRCCS Policlinico S. Matteo, Pavia, Italy; 4 Istituto Scientifico Romagnolo per lo Studio e la Cura dei Tumori (IRST)- IRCCS, Osteoncology and Rare Tumors Center, Meldola, Italy; 5 Treviglio and Caravaggio Hospital, Division of Medical Oncology, Treviglio, Italy; 6 Division of Medical Oncology 2, Azienda Ospedaliero-Universitaria Pisana, Istituto Toscano Tumori, Pisa, Italy; 7 Medical Oncology, Department of Clinical and Biological Sciences, A.O.U. San Luigi Gonzaga, Orbassano, Italy; 8 Clinica di Oncologia Medica, A. O. Ospedali Riuniti-Universitá Politecnica delle Marche, Ancona, Italy; 9 Ospedale Mauriziano Umberto I di Torino e Istituto Per La Ricerca e La Cura del Cancro di Candiolo, Torino, Italy; 10 Department of Medical Oncology, Presidio Sanitario Gradenigo, Turin, Italy; 11 Azienda Ospedaliero di Rilievo Nazionale A. Cardarelli, Naples, Italy; 12 INI, Grottaferrata, Italy; 13 Medical Oncology Unit – University of Ancona, Ancona, Italy; 14 Medical Oncology Unit - IRCCS National Cancer Institute “Giovanni Paolo II”, Bari, Italy; 15 S.C. Oncologia Medica 1, C.O.E.S. - Centro Oncologico ed Ematologico Subalpino, Azienda Ospedaliero Universitaria San Giovanni Battista di Torino, Molinette, Italy; 16 Division of Medical Oncology, Hospital of Legnano, Milan, Italy; 17 Section of Medical Oncology, Department of Surgery and Oncology, University of Palermo, Palermo, Italy; 18 U.O.C. Oncologia, Azienda Ospedaliera “Ospedali Riuniti Marche Nord”, Presidio San Salvatore, Pesaro, Italy; 19 Department of Medical Oncology, University of Cagliari, Cagliari, Italy; 20 Unit Integrated Therapies in Oncology, Department of Human Pathology, University of Messina, Messina, Italy; 21 Oncology Unit, IRCCS Casa Sollievo della Sofferenza, San Giovanni Rotondo (FG), Italy; 22 Divisione di Oncologia, Dipartimento Integrato di Oncologia ed Ematologia, Università degli Studi di Modena e Reggio Emilia, Italy; 23 Department of Medical Oncology, Regina Elena National Cancer Institute, Rome, Italy; 24 Medical Oncology Department & Breast Unit - Hospital of Brindisi and Medical Oncology Department - European Institute of Oncology, Milan, Italy; 25 Department of Oncology, USL-8, Ospedale San Donato, Arezzo, Italy; Van Andel Institute, United States of America

## Abstract

**Background:**

Bone metastasis represents an increasing clinical problem in advanced renal cell carcinoma (RCC) as disease-related survival improves. There are few data on the natural history of bone disease in RCC.

**Patients and methods:**

Data on clinicopathology, survival, skeletal-related events (SREs), and bone-directed therapies for 398 deceased RCC patients (286 male, 112 female) with evidence of bone metastasis were statistically analyzed.

**Results:**

Median time to bone metastasis was 25 months for patients without bone metastasis at diagnosis. Median time to diagnosis of bone metastasis by MSKCC risk was 24 months for good, 5 months for intermediate, and 0 months for poor risk. Median number of SREs/patient was one, and 71% of patients experienced at least one SRE. Median times to first, second, and third SRE were 2, 5, and 12 months, respectively. Median survival was 12 months after bone metastasis diagnosis and 10 months after first SRE. Among 181 patients who received zoledronic acid (ZOL), median time to first SRE was significantly prolonged versus control (*n* = 186) (3 months vs 1 month for control; *P*<0.05).

**Conclusions:**

RCC patients with bone metastasis are at continuous risk of SREs, and in this survey ZOL effectively reduced this risk.

## Introduction

Kidney cancer is one of the 10 most frequently occurring cancers in the Western world. More than 250,000 new cases of kidney cancer are diagnosed annually and 116,000 patients die from the disease [1]. Renal cell carcinoma (RCC) accounts for 80%–90% of kidney cancers. Almost one third of patients present with metastatic disease and another 20% experience recurrence and develop metastatic RCC after nephrectomy [2], [3]. Despite advances in systemic therapy and increases in survival over the past decade, median survival in metastatic RCC remains low, at 19.7 months [4].

Bone metastasis has been identified as an independent prognostic variable associated with poor survival in patients with metastatic RCC [5]. With disease progression, approximately 30% of RCC patients develop bone metastasis [6], representing the second most common site of distant metastatic spread (after lung) in advanced RCC. Bone metastases in RCC are mainly osteolytic in nature, and decrease bone integrity, induce bone pain, and result in significant morbidity for patients from the associated skeletal-related events (SREs) [7], defined as pathologic fractures, the need for radiotherapy for bone pain, surgical interventions to treat or prevent an impending fracture, spinal cord and nerve root compressions, and hypercalcemia. Skeletal-related events cause significant decreased functional independence and loss of autonomy and impair patients' quality of life [8]. Radiotherapy is the most common SRE in RCC patients: approximately 81% of patients with RCC receive radiotherapy, 42% develop long-bone fracture, and 29% require orthopedic surgery [7]. Despite the fact that bone metastasis from RCC causes higher rates of SREs compared with many other tumors [9], metastatic bone disease in renal cancer has received little attention to date. Previous studies of the burden of bone disease in solid tumors included smaller numbers of patients with RCC (ranging between 46 and 254) [6], [7], [10]–[12], and the risk factors for SREs in these patients need further elucidation. However, one study demonstrated a correlation between elevated levels of an osteolysis marker, N-telopeptide of type I collagen (NTX), and overall survival (*P* = 0.0001) [12]. Nonetheless, there remains a paucity of data regarding the demographics of bone metastasis and subsequent SREs in RCC patients. Early detection and availability of novel primary therapies are extending patient survival, thereby leaving patients with bone metastasis at risk of SREs for longer durations. Thus, understanding the natural history of bone metastases (and their clinical management) for RCC in the “era” of targeted therapies is of increasing importance for reducing healthcare costs and improving patients' quality of life. Here we report final data from a large Italian multicenter study of patients with bone metastasis from RCC.

## Methods

### Study Design

This was a retrospective, observational multicenter study of medical records from 1986 to 2011 for patients diagnosed with RCC who were treated at 23 different centers in Italy. Data were collected from patients of all ages who received standard treatments (i.e. not on clinical trials or experimental protocols) in accordance with their treating physician's practice. Only patients with RCC who had at least one bone metastasis during the course of their disease and who died of RCC or RCC-related complications were included in the study. All patients were deceased at the time of analysis. Patients were identified as having bone metastasis if two of the following criteria were satisfied: physician reported bone metastasis; bone metastasis identified on bone scan; record of radiotherapy to bone as a palliative measure; identification of bone metastasis by other imaging assessment (e.g. standard x-rays, computed tomography scans, or magnetic resonance imaging of the skeleton).

Data were collected throughout the disease course for all cancer treatments, including surgery, radiation therapy, chemotherapy, and targeted therapies. Variables assessed included age, sex, histological subtype, number and sites of bone metastasis, Memorial Sloan-Kettering Cancer Center (MSKCC) risk score, time to appearance of bone metastasis, presence of bone pain, times to first and subsequent SREs (from diagnosis of bone metastasis), SRE types, survival after first SRE, and type and times of bisphosphonate therapy. Patients, when possible, had documented bone pain scores on a visual analogue scale of 0–10.

This multicenter retrospective observational study has been approved by the Ethics Committee of the coordinator center (University Campus Bio-Medico of Rome). According to Ethics Committee, a written consent was not needed. In fact, this is a study considering only died patients whose recruitment in the analysis did not influenced their treatment.

### Statistical Analysis

Descriptive statistics were used for patient demographics and incidence of SREs. All survival intervals were determined by the Kaplan-Meier method. Differences in median time to first SRE were evaluated by log-rank test. SPSS software (version 14.00; SPSS, Chicago, IL) was used for statistical analysis. A *P* value<0.05 was considered statistically significant.

## Results

### Patient Characteristics

After reviewing the records of more than 1800 patients who died from RCC, 398 patients with bone metastasis were identified: 124 (31%) had bone metastasis at the time of RCC diagnosis and 269 (68%) developed bone metastasis after RCC diagnosis. Time of development of bone metastasis relative to RCC diagnosis was not determined for five patients. Of these 398 patients, 286 (72%) were male (Table 1), consistent with the known male predominance of RCC [1]. The median age was 63 years. Regarding bisphosphonate use, 45% were treated with zoledronic acid (ZOL), 8% received pamidronate, and 47% did not receive any bisphosphonate treatment. Tumor histology was predominantly clear cell (Table 1).

**Table 1:** Baseline Patient Demographics.

Characteristic	Patients, n (%) (*N* = 398)
**Median age, y (range)**	63 (16–92)
**Sex**	
Male	286 (72)
Female	112 (28)
**Tumor histotype**	
Clear cell	345 (87)
Papillary	22 (6)
Chromophobe	3 (<1)
Other	16 (4)
Not available	12 (3)
**Number of bone metastases**	
1	116 (29)
≥2	282 (71)
**Location of lesion**	
Spinal column	271 (68)
Limb	155 (39)
Long bone	125 (31)
Other	68 (17)
**Lesion type**	
Osteolytic	316 (79)
Osteoblastic	27 (7)
Mixed	52 (13)
Unknown	3 (<1)

### Skeletal Metastases

**Figure 1:**
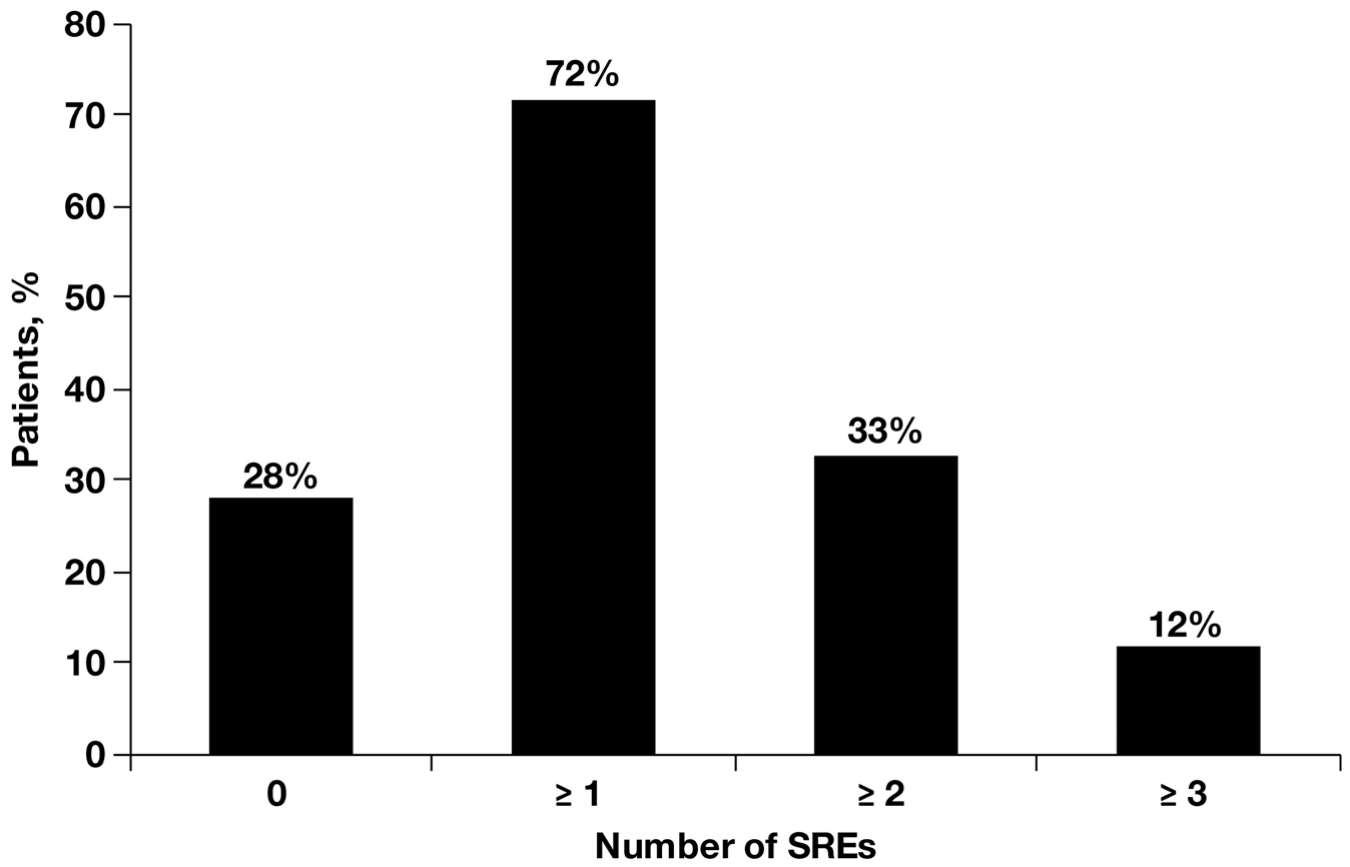
Skeletal-related events (SREs) are common in patients with bone metastasis from renal cell carcinoma (*N* = 398).

**Figure 2:**
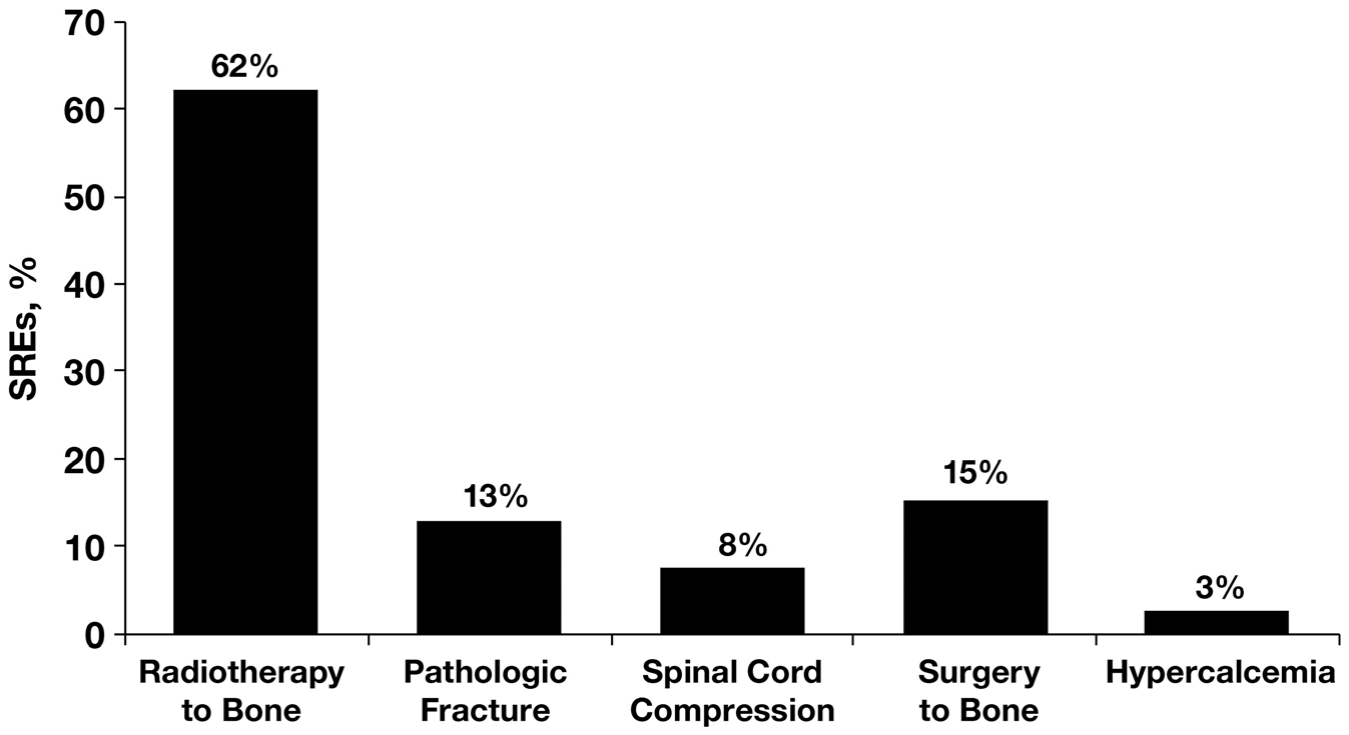
Incidence of skeletal-related events (SREs) in patients with bone metastases from renal cell carcinoma (*N* = 398).

Most patients (281 [71%]) had multiple bone metastases and 116 (29%) had a single bone metastasis. The spinal column was the most common site of bone metastasis (68% of patients). Osteolytic lesions (79%) were far more prevalent in this group than mixed (13%) or osteoblastic (7%) lesions (Table 1). The majority of the patients (72%) experienced at least one SRE, 33% experienced at least two SREs, and 12% experienced at least three SREs (Figure 1). The incidences of different SREs (Figure 2) were consistent with earlier reports [13], with radiotherapy to bone being the most common SRE (62% of all events), followed by surgery to bone, which accounted for 15% of the total number of SREs experienced in this analysis.

### Skeletal Outcomes and SREs

In patients without bone metastasis at primary diagnosis of RCC (*N* = 269), the median time to diagnosis of bone metastasis was 25 months (range, 1–288 months). The median time to the appearance of bone metastasis in the overall population was 8 months (range, 0–288 months). The median level of maximum bone pain experienced after diagnosis of bone metastasis was 7 (range, 0–10). The median pain level experienced at the time of diagnosis of bone metastasis was 4 (range, 0–9).

The median number of SREs experienced by patients was one (range, 0–6). The median time to first SRE after confirmed diagnosis of bone metastasis was 2 months (range, 0–72 months), indicative of the aggressiveness of bone metastasis in RCC. The median time to second SRE was 5 months (range, 0–113 months), and to third SRE was 12 months (range, 1–108 months). Median survival from the diagnosis of bone metastasis was 12 months (range, 1–178 months). Median survival after development of the first SRE was 10 months (range, 0–144 months). More than 70% of the patients experienced at least one SRE, and the median survival in these patients was 14 months (range, 1–178 months). Intriguingly, median survival in patients who did not experience SREs (∼30% of the study population) was only 9 months (range, 0–62 months). Although the precise reasons for this are not known, it is likely that these patients had rapidly progressing visceral metastases and correspondingly shorter survival.

**Figure 3:**
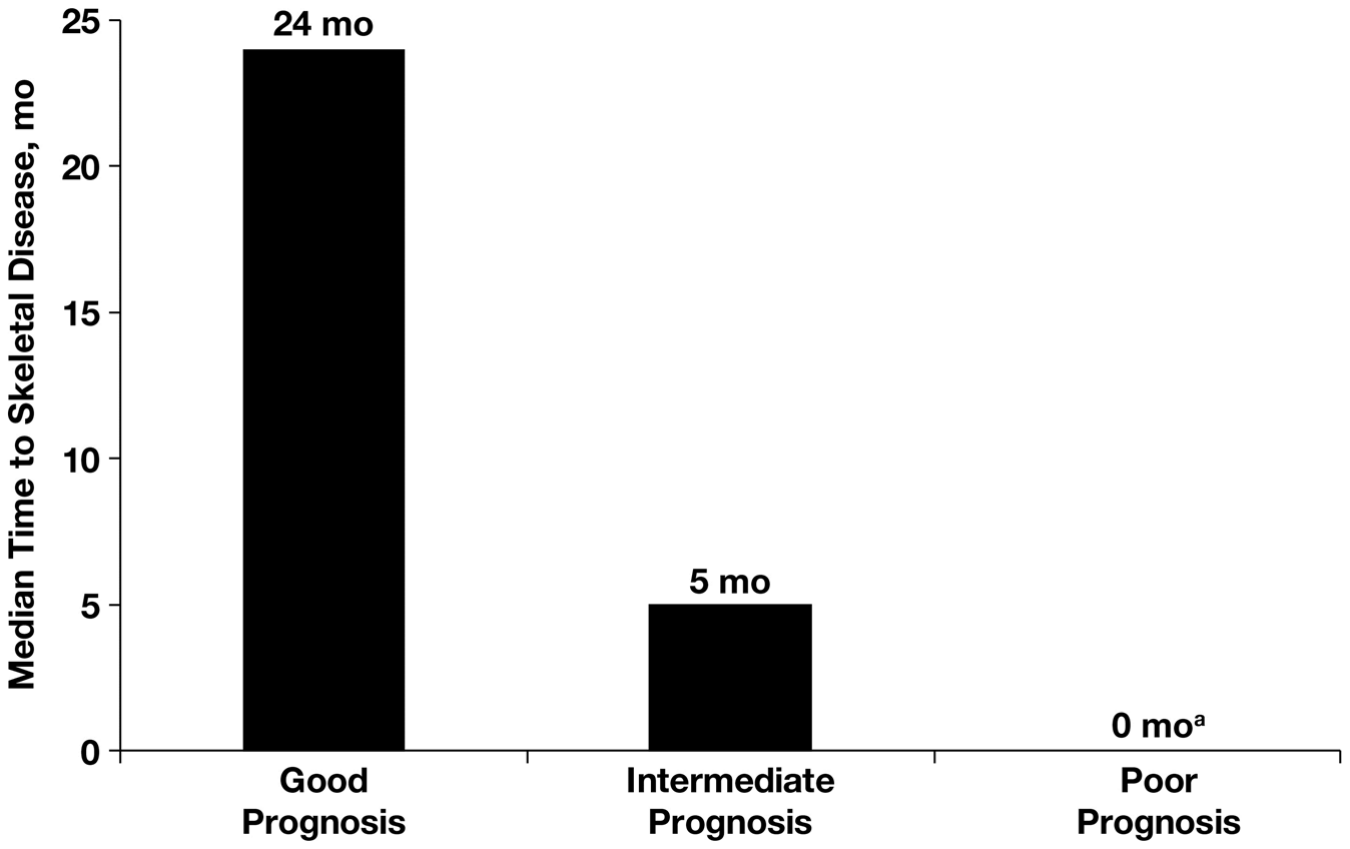
Median time to bone metastases diagnosis according to MSKCC risk score. ^a^ Significant correlation between poor prognosis by MSKCC risk score and median time to bone metastasis (*P*<0.05). MSKCC, Memorial Sloan-Kettering Cancer Center.

**Figure 4:**
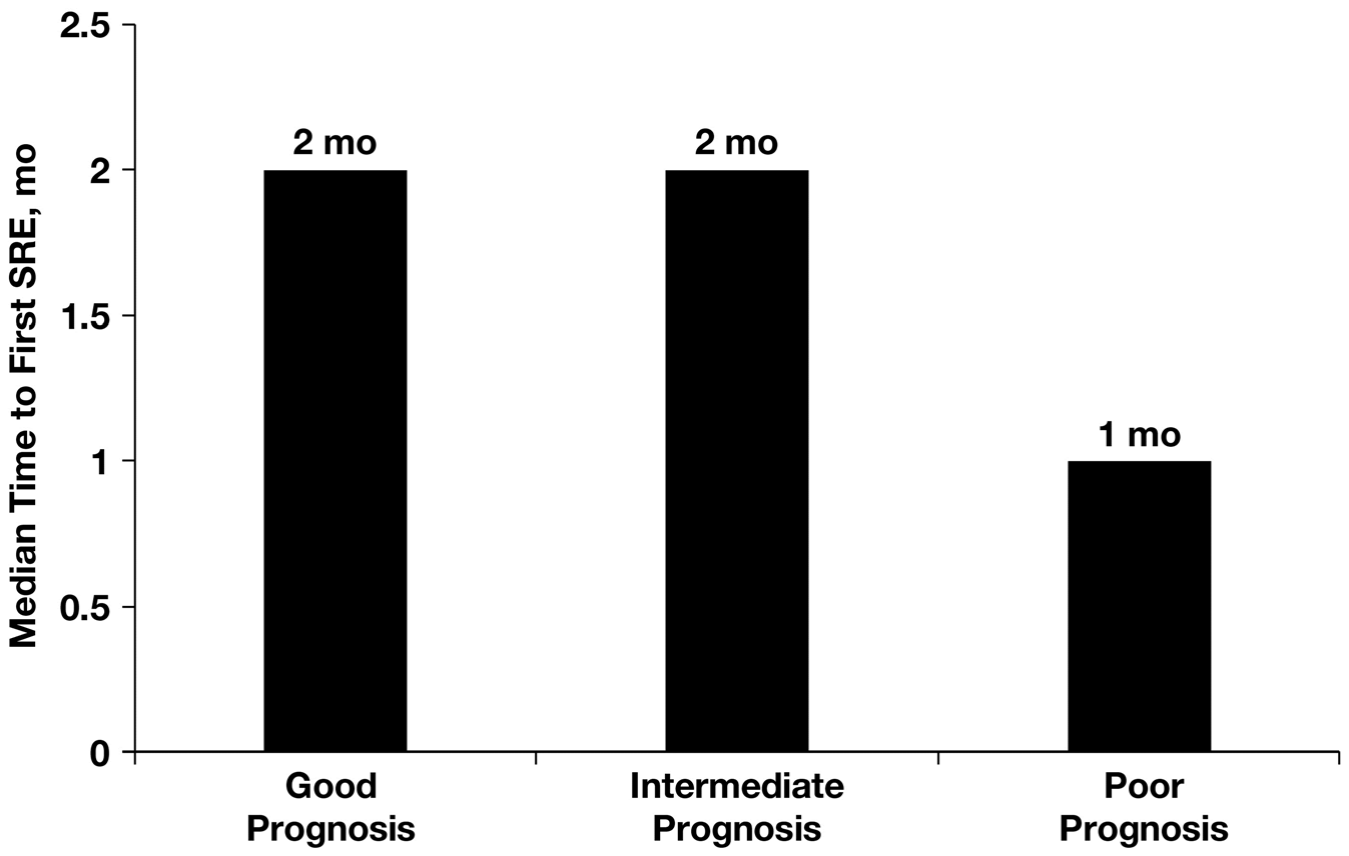
Median time to first skeletal-related event (SRE) according to MSKCC risk score. MSKCC, Memorial Sloan-Kettering Cancer Center.

Bone metastasis diagnosis also correlated with MSKCC risk: median time to bone metastasis diagnosis in the good risk group was 24 months (range, 0–288 months) versus 5 months (range, 0–265 months) in the intermediate risk group and 0 months (range, 0–77 months) in the poor risk group (*P*<0.05; Figure 3). Time to first SRE also correlated with MSKCC risk, albeit to a lesser extent: median time to first SRE in the good risk group was 2 months (range, 0–72 months), which was similar to that in the intermediate risk group (2 months [range, 0–26 months]) but higher than that in the poor risk group (1 month [range, 0–25 months]) (Figure 4).

### Bisphosphonate Therapy

**Figure 5:**
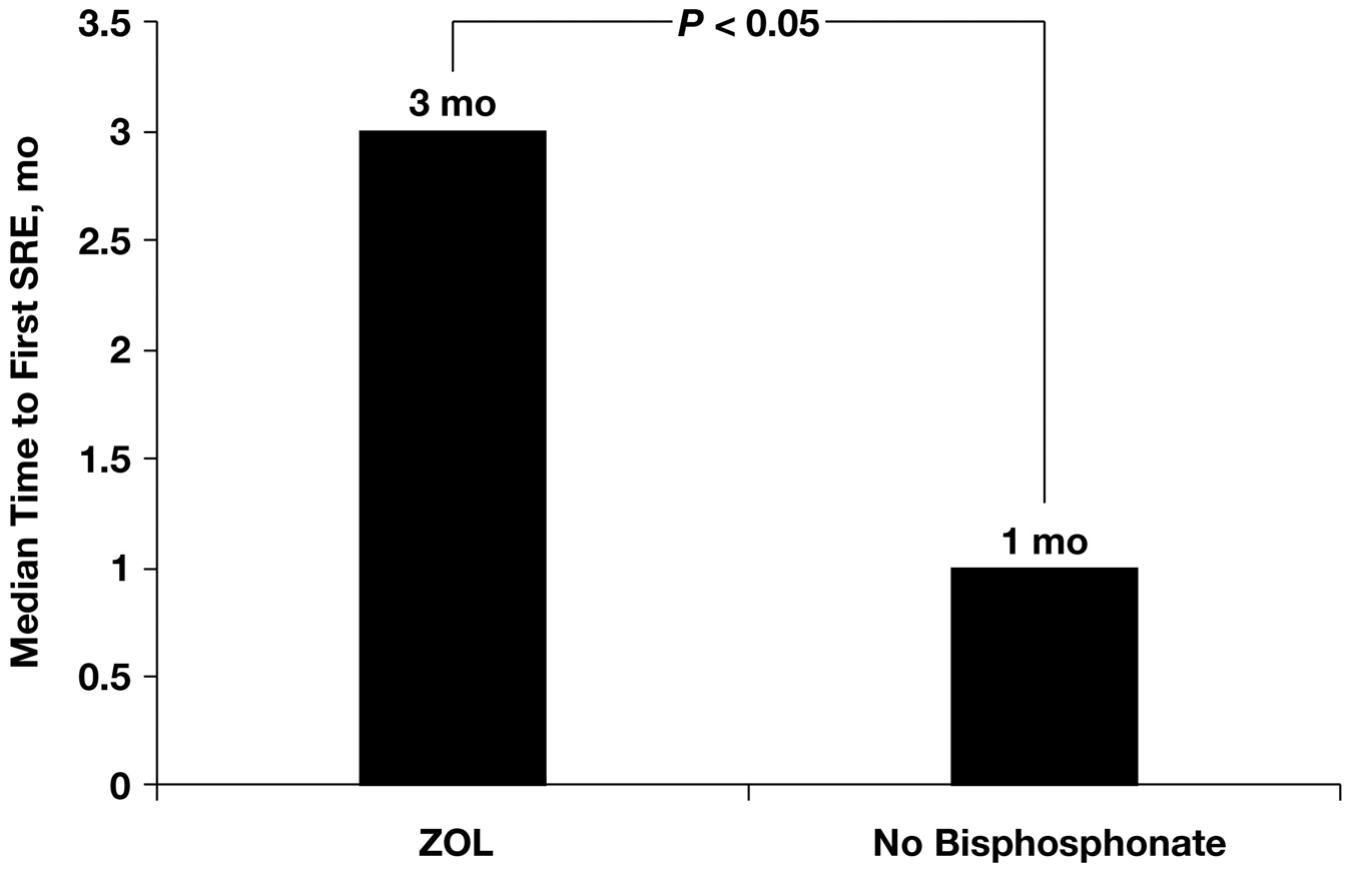
Median time to first skeletal-related event (SRE) in renal cell carcinoma patients receiving ZOL (*n* = 181) and those who did not receive bisphosphonate treatment (*n* = 186). ZOL, zoledronic acid.

Among the 398 patients with bone metastasis, 186 (47%) did not receive any bisphosphonate treatment, 181 patients (45%) received ZOL (administered at a dose of 4 mg every 4 weeks via 15-minute infusion, with dose adjustments based on creatinine clearance), and 31 patients (8%) received pamidronate (administered at a dose of 90 mg every 4 weeks via 2-hour infusion). Zoledronic acid was generally well tolerated; six patients (1.5%) developed osteonecrosis of the jaw (ONJ). Patients with ONJ underwent a computed tomography scan for confirmation; no retrospective adjudication was performed. It should be noted that no preventive dental care was offered because many patients received treatment before 2004. Patients receiving ZOL treatment had a longer median survival time from diagnosis of bone metastasis compared with patients not treated with bisphosphonates (15 months [range, 2–120 months] versus 7 months [range, 1–178 months], respectively) (*P*<0.05; Table 2). Patients who received ZOL also had a significant delay in time to first SRE from diagnosis of bone metastasis versus patients who did not receive bisphosphonate treatment (3 months [range, 0–101 months] versus 1 month [range, 0–22 months], *P*<0.05) (Figure 5).

**Table 2:** Median Survival after Bone Metastases Diagnosis.

Variable	Time, mo	Range
**All patients**	12	1–178
**By SRE history**		
Patients with at least one SRE	14	1–178
No SRE	9	0–62
After first SRE	10	0–144
**By bisphosphonate treatment**		
No bisphosphonate	7	1–178
ZOL treated	15	2–120

SRE, skeletal-related event; ZOL, zoledronic acid.

## Discussion

To our knowledge, this study is the largest multicenter survey to investigate the natural history of metastatic bone disease in patients with RCC. Bone metastasis was confirmed in approximately 22% of our screened RCC patients, which is lower than previous estimates of bone metastasis in approximately 30% of patients [6], [7]. In the present study only patients with at least one known bone metastasis were included, and several patients with poorly documented bone metastasis were omitted. This might explain the slightly lower incidence rate of bone metastasis in the patient population of this study.

Among the 22% of RCC patients with bone metastasis, approximately one third presented with bone metastasis at the time of initial RCC diagnosis, whereas the remainder developed bone metastasis during disease progression. This is in contrast with a previous study by Woodward et al. [6], wherein an almost equal distribution was reported between patients having bone metastasis at the time of primary diagnosis and patients developing bone metastasis at later times, which may be due to differences in screening practices as well as the limits of bone metastasis detection between studies. The axial skeleton was involved in 68% of our RCC patients, which is lower than the 83% frequency observed in breast cancer [14]. However, sites of metastatic growth may be governed by the mechanism of metastasis. In RCC, the majority of metastatic sites are found in the pelvis, sacrum, and lumbar spine, although metastasis to the long bones is not uncommon [15]. In fact, in one RCC study (*N* = 31), 33% of patients were found to have metastases in their femur or humerus [16].

The median survival time of 12 months after diagnosis of skeletal metastasis in our study population is consistent with previously reported median survival times in patients with bone metastasis from RCC. Median survival was paradoxically shorter in patients with no SREs versus those with at least one SRE (9 months vs 14 months), possibly because of aggressive visceral metastases affecting survival, or other complications they experienced.

Bone metastasis has been identified as an independent prognostic variable associated with poor survival in patients with metastatic RCC [5]. In their skeletal history, the majority of these patients may experience extremely debilitating skeletal complications (i.e. SREs) that profoundly impact their quality of life [8], thereby highlighting the need for effective bone-targeted therapy. Given the increasing incidence of RCC, improvements in overall survival over the last decade [17], and the higher rates of SRE occurrence in RCC patients compared with other cancer patients [9], a better understanding of the natural history of bone metastasis in RCC may provide insight into appropriate monitoring for SREs.

Traditional cytokines or immunotherapy (interferon alfa or interleukin-2) might have been used for some patients in our retrospective analysis, but are not very effective in treating metastatic bone disease [5]. In the past 6 years, widespread use of targeted therapies directed at the vascular endothelial growth factor (VEGF) and mammalian target of rapamycin (mTOR) signaling pathways [18], [19] has greatly changed the clinical management of metastatic RCC. Since 2005, six molecular-targeted agents have been approved in the United States and Europe for treating patients with advanced or metastatic RCC: sorafenib, sunitinib, bevacizumab (in combination with interferon alfa), temsirolimus, everolimus, and pazopanib [19], [20]. The progression-free survival and overall survival times achieved with these targeted agents are substantially superior to those of cytokine-based therapies; however, there is no evidence for greater efficacy of newer targeted therapies against bone disease in particular (although recent data suggest that bisphosphonates may augment the effects of sunitinib in this clinical setting) [17]. Bisphosphonates (such as ZOL, pamidronate, and clodronate) are highly effective inhibitors of osteoclast-mediated bone resorption and have been widely used for treating and preventing SREs from bone metastases of breast cancer and prostate cancer, and from bone lesions of multiple myeloma [21]–[24]. It is important to note that although ZOL, pamidronate, and clodronate are all approved for use in patients with bone metastasis from breast cancer or bone lesions from multiple myeloma, ZOL is the only bisphosphonate with approved efficacy in the RCC setting. More recently, the receptor activator of nuclear factor kappa-B ligand inhibitor denosumab has also shown broad efficacy for SRE reduction in patients with bone metastasis from solid tumors [25]; however, denosumab was not available outside of a clinical trial during the period spanned by our retrospective database analysis.

A subanalysis of 74 RCC patient groups from a phase III, placebo-controlled study showed that ZOL can significantly delay the onset of SREs (*P* = 0.006) and provide a numeric increase in median overall survival in these patients [13]. Our larger study, which included 181 patients treated with ZOL, showed similar results, with a significant extension of time to first SRE and increase in the median survival time after diagnosis of bone metastasis. Taken together, these data support the beneficial effects of ZOL in RCC patients. Additionally, although intravenous bisphosphonates have been associated with dose- and infusion rate-dependent decreases in renal function [26], in the current study the renal safety profile of ZOL in RCC was similar to the renal safety profile in patients not treated with bisphosphonates.

Limitations of this study include its retrospective design and inclusion of an unselected heterogeneous cohort of patients with all types of histologic variants of RCC, as well as a range of anticancer therapies. However, the types of patients included in this study represent the typical scenario of a real clinical practice. Another limitation of a chart review is the heterogeneity of standardized methods used for detecting bone metastases, with each methodology having its own limit of detection. Accurate and early identification of patients with bone metastasis is crucial for initiating treatment and delaying onset of SREs.

Early detection of bone metastasis is essential for optimal management and treatment of SREs in patients with metastatic RCC. With the advent of targeted therapies, it is likely that patients will survive longer with an increased chance of developing bone metastasis and SREs. To our knowledge, this retrospective analysis is the largest multicenter study to demonstrate that bone metastases from RCC are commonly aggressive and result in relatively early onset of SREs in the majority of patients. This retrospective analysis provides further support that ZOL is effective for reducing the incidence of SREs in patients with bone metastasis from RCC.
